# En-bloc resection of a giant chest wall tumour and skeletal reconstruction with methyl methacrylate in a resource poor environment: case report

**DOI:** 10.1186/1749-8090-8-S1-O247

**Published:** 2013-09-11

**Authors:** U Abubakar, M Danfulani, B Kakale, IR Jamalu

**Affiliations:** 1 Cardiothoracic Surgery Unit, Department of Surgery, Usmanu Danfodiyo University Teaching Hospital, Sokoto, Nigeria; 2 Department of Radiology, Usmanu Danfodiyo University Teaching Hospital, Sokoto, Nigeria; 3 Department of Surgery, Usmanu Danfodiyo University Teaching Hospital, Sokoto, Nigeria

## 

We describe a 42 year old man who presented to us with a left lateral chest wall mass of 2years duration. It has been increasing in size since it was noticed, initially painless but became painful 2 months prior to presentation. No history of trauma to the chest and no cough or haemoptysis. He is a known hypertensive regular on his medications.

Examination revealed a left lateral chest wall mass, firm in consistency, non-tender, measuring 26x18x10cm. He had reduced air entry on the left middle and lower lung zones.

Chest X-ray shows a left lateral soft tissue mass with homogenous opacity involving the left middle and lower lobes. Chest CT scan shows a left soft tissue mass with intrathoracic extension and destruction of the 4th and 5th ribs. There is also compressive atelectasis of the left middle and lower lobes. No features suggestive of underlying lung pathology.

He had en-bloc resection of the chest wall tumour and skeletal reconstruction with methyl metacrylate” sandwich” in prolene mesh. Postoperative recovery was uneventful and was discharged on the 14th day post-op. Histology of the tumour revealed a neurofibroma.

We still see patients with giant chest wall tumours in this part of the world presenting only when the tumours are symptomatic. Even though these tumours can be huge, curative resection and skeletal reconstruction can be offered with good outcome.

